# The role of cangrelor in acute and high-risk PCI settings

**DOI:** 10.1093/ehjcvp/pvaf042

**Published:** 2025-06-05

**Authors:** Uwe Zeymer, Tobias Geisler, Dirk Westermann, Kurt Huber

**Affiliations:** Institut für Herzinfarktforschung Ludwigshafen, Bremserstrasse 79, Ludwigshafen 67063, Germany; Faculty of Medicine, University Heart Center Freiburg-Bad Krozingen, University of Freiburg, Südring 15, Bad Krozingen 79189, Germany; Department of Cardiology, University Hospital Tübingen, Tübingen 72076, Germany; Faculty of Medicine, University Heart Center Freiburg-Bad Krozingen, University of Freiburg, Südring 15, Bad Krozingen 79189, Germany; Department of Cardiology, Wilhelminen Hospital, Vienna 1090, Austria; Sigmund Freud University, Vienna 1020, Austria

**Keywords:** Cangrelor, STEMI, Cardiogenic Shock, Cardiac Arrest, High-risk PCI, P2Y_12_ inhibitor

## Abstract

Dual antiplatelet therapy with acetylsalicylic acid (ASA) and an oral P2Y_12_ inhibitor is the standard of care to prevent thrombotic complications in patients with acute coronary syndromes undergoing percutaneous coronary intervention (PCI). However, the oral administration of P2Y_12_ inhibitors bears significant limitations in acute and high-risk PCIs, particularly in ST-elevation myocardial infarction (STEMI) patients, especially those presenting with cardiogenic shock (CS) and cardiac arrest (CA). In these cases, factors such as active vomiting, altered physiology, sedatives, mechanical ventilation, and therapeutic hypothermia can impair drug absorption, reducing the intended antiplatelet effect and increasing ischaemic risk. In these cases, intravenous antiplatelet strategies with ASA and cangrelor could guarantee adequate periprocedural platelet inhibition. Here, we discuss the role of cangrelor in acute and high-risk PCI settings. The pharmacokinetic and pharmacodynamic attributes of cangrelor are discussed first, underscoring the distinctive features that make cangrelor an attractive antiplatelet agent in acute PCI settings. The second part of the review summarizes the evidence from real-world studies that illustrate how cangrelor has been adopted in contemporary practice. Finally, we provide a practical guide to cangrelor use, including recommendations for transitioning from cangrelor to oral P2Y_12_ inhibitors after PCI.

## Graphical Abstract

**Graphical Abstract:**
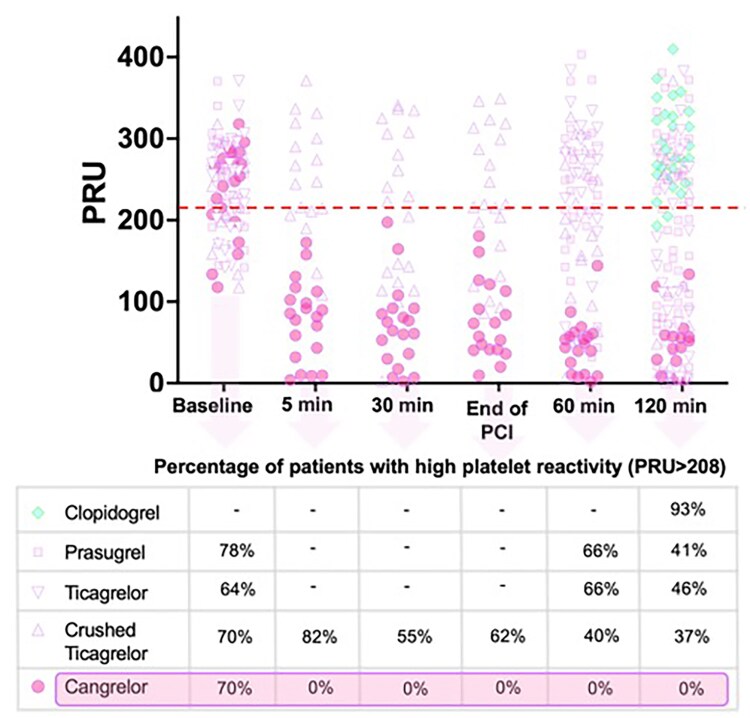

## Introduction

Effective platelet inhibition reduces the risk of ischaemic events in patients with acute coronary syndromes (ACS) undergoing percutaneous coronary intervention (PCI).^1,2^ Periprocedural antiplatelet strategies aim to inhibit the activation and aggregation of platelets to prevent thrombus formation, stent thrombosis (ST), and myocardial infarction (MI).^3^

Dual antiplatelet therapy (DAPT) with acetylsalicylic acid (ASA) and a P2Y_12_ inhibitor (clopidogrel, prasugrel, ticagrelor, or cangrelor) is the standard of care to prevent thrombotic complications in ACS patients treated with PCI.^1,2^ The routine use of Glycoprotein IIb/IIIa (GPIIb/IIIa) inhibitors (abciximab, eptifibatide, and tirofiban) is no longer recommended due to the increased risk of bleeding associated with their use, and they are reserved for bailout situations.^1,2^

Pre-treatment of ACS patients with oral P2Y_12_ inhibitors and ASA before PCI has been routine practice for many years.^4–7^ Nevertheless, randomized trials have failed to establish the advantage of pre-treatment with oral P2Y_12_ inhibitors in non-ST-elevation MI (NSTEMI),^8,9^ while there is a reduction in ischaemic events in ST-elevation MI (STEMI).^10^ The most recent 2023 European Society of Cardiology (ESC) Guidelines for the Management of ACS do not recommend pre-treatment with oral P2Y_12_ inhibitors in NSTEMI (Class III) and downgraded pre-treatment in STEMI from Class I (‘recommended’) to Class IIb (‘may be considered’).^2^ According to the 2025 ACC/AHA/ACEP/NAEMSP/SCAI Guideline for Coronary Artery Revascularization, routine pre-treatment with oral P2Y_12_ inhibitors in patients undergoing an early invasive management strategy (<24 h) is not supported by clinical trial data.^1^

The oral route of administration has significant limitations in acute ACS settings, particularly in STEMI cases complicated by cardiogenic shock (CS) and cardiac arrest (CA), where rapid P2Y_12_ inhibition is crucial. In such settings, compromised systemic absorption, active vomiting, altered physiology,^11,12^ the use of sedatives (e.g. morphine or fentanyl),^13–16^ difficulty in swallowing due to mechanical ventilation, or therapeutic hypothermia^17^ hamper the intended antiplatelet effect of oral P2Y_12_ inhibitors, resulting in an increased, and often unnoticed, ischaemic risk. In these situations, systemic absorption of oral P2Y_12_ inhibitors may require 2–6 h from their oral uptake in STEMI,^18–21^ far exceeding the average duration of contemporary PCI procedures (<60 min).^20,22^ Consequently, in P2Y_12_ inhibitor-naive patients requiring urgent PCI, intravenous antiplatelet strategies, such as DAPT with intravenous ASA and cangrelor, are the only options ensuring adequate periprocedural platelet inhibition.^20,21,23^

In this review, we discuss the role of cangrelor in acute and high-risk PCI settings. The pharmacokinetic (PK) and pharmacodynamic (PD) profile of cangrelor is discussed first, highlighting the key features that make cangrelor a valuable antiplatelet agent in acute PCI settings compared to oral P2Y_12_ inhibitors and GPIIb/IIIa inhibitors. The second part of the review summarizes the evidence from real-world studies that illustrate how cangrelor has been adopted in contemporary practice. We also provide a practical guide to cangrelor use, including recommendations for transitioning from cangrelor to oral P2Y_12_ inhibitors after PCI.

## Cangrelor: Effective platelet inhibition/profile for acute PCIs

Cangrelor is an adenosine triphosphate analogue that is rapidly dephosphorylated in plasma and selectively blocks the P2Y_12_ receptor in a direct, reversible, and competitive manner.^24,25^ Cangrelor reaches the maximum plasma concentration within 2 min and has a linear dose-dependent pharmacokinetic profile.^26^ The mean steady-state concentration of cangrelor during a constant intravenous infusion of 4 micrograms/kg/min is 488 ng/mL, which provides profound platelet inhibition for the duration of the infusion.^26^ The offset of cangrelor refers to its short half-life of 2–3 min, with platelet function returning to normal levels within 30 min to an hour after discontinuation.^26^

### Cangrelor compared with oral P2Y_12_ inhibitors

The PK/PD features of cangrelor and the oral P2Y_12_ inhibitors are shown in *Table 1* (first four rows, adapted from Angiolillo *et al.*^27^). Clopidogrel and Prasugrel are thienopyridine prodrugs that require conversion of the parent drug into the active metabolite.^27^ The active metabolites of clopidogrel and prasugrel irreversibly bind the adenosine diphosphate (ADP)-binding site of the P2Y_12_ receptor, rendering the receptor non-functional for the life of the platelet.^28^ Accordingly, the offset of clopidogrel and prasugrel is the longest among P2Y_12_ inhibitors and refers to the turnover of platelets (up to 10 days). Ticagrelor binds to the P2Y_12_ receptor in a site distinct from the ADP receptor through a non-competitive, reversible, and allosteric mechanism.^28^ Ticagrelor does not require metabolic activation and has a shorter offset of action than thienopyridines, which bind irreversibly to the P2Y_12_ receptor. Clopidogrel and ticagrelor display Cytochrome P450 (CYP) interaction, which potentially could give rise to drug interactions.

**Table 1:** Overview of the available P2Y_12_ inhibitors

	FDA/EMA approval	Drug class	Administration route	Prodrug	CYP drug interaction	Onset of action	Receptor blockade	Half-life of the parent drug	Half-life (active metabolite)	Offset of action	Renal adjustment
Clopidogrel	1997/2008	P2Y12 inhibitor	Oral	Yes	CYP2C19	2–8h	Irreversible	6h	30 min	5–10 days	No
Prasugrel	2007/2009	P2Y12 inhibitor	Oral	Yes	No	30 min-4h	Irreversible	<5 min	30–60 min	7–10 days	No
Ticagrelor	2011/2010	P2Y12 inhibitor	Oral	No	CYP3A	30 min-4h	Reversible	6–12h	–	3–5 days	No
Cangrelor	2015/2015	P2Y12 inhibitor	Intravenous	No	No	immediate	Reversible	3–6 min	–	60 min	No
Eptifibatide	1998/1999	GPIIb/IIIa inhibitor	Intravenous	No	No	immediate	Reversible	2–2.5h	–	6–8h	Yes
Tirofiban	1998/1999	GPIIb/IIIa inhibitor	Intravenous	No	No	immediate	Reversible	2h	–	2–4h	Yes

CYP, Cytochrome P450; GPIIb/IIIa, glycoprotein IIb/IIIa; EMA, European Medicines Agency; FDA, Food and Drug Administration.

The onset of action of oral P2Y_12_ inhibitors shows interpatient variability. The onset of clopidogrel is typically between 2 to 8 h and between 30 min and 4 h for prasugrel and ticagrelor.^27^ Notably, a significant proportion of patients (20%–40%) exhibit varying degrees of clopidogrel resistance, depending on whether they carry loss-of-function of a single (intermediate metabolizers) or both alleles (poor metabolizers) of the CYP2C19 gene, which is essential for converting clopidogrel into its active metabolite.^29^

Several factors can compromise the pharmacological effect of oral P2Y_12_ inhibitors in acute PCI settings, such as poor systemic absorption, delayed onset of action, and baseline platelet aggregability. One study showed that the impaired response to ticagrelor in STEMI patients could not be overcome by increases in the loading dose up to 360 mg.^30^ The action of oral P2Y_12_ inhibitors can be further delayed when sedatives are used for pain management, as they reduce gastric and intestinal motility and, therefore, systemic absorption. Pharmacologic studies have shown a lower antiplatelet efficacy of oral P2Y_12_ inhibitors in ACS patients treated with morphine or fentanyl, irrespective of the oral P2Y_12_ used. Alternative strategies with crushed tablets and orodispersible formulations have been developed to improve their systemic absorption and enable administration in unconscious and intubated patients.^20,31–33^ Crushed ticagrelor and prasugrel are feasible in STEMI and provide somewhat earlier platelet inhibition than integral tablets. Nevertheless, the systemic absorption of crushed pills still requires 30–60 min and is still affected by the concomitant use of sedatives.^20,31^

**Figure 1:**
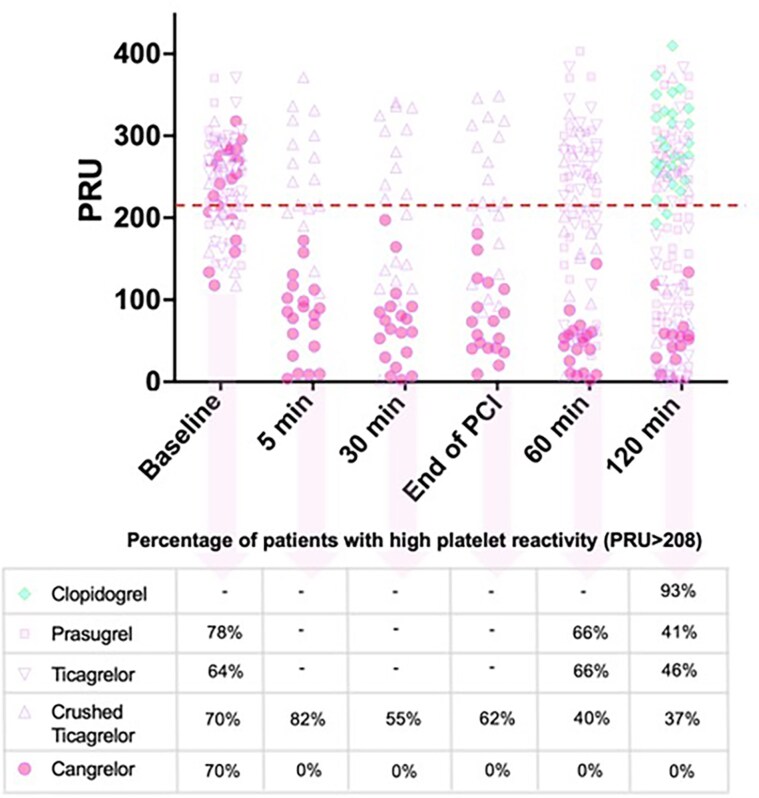
Platelet reactivity units (PRU) at different time points for clopidogrel, prasugrel, ticagrelor (integral and crushed), and cangrelor. Data points were extracted using automeris.io (https://automeris.io) from three independent studies that measured platelet reactivity in ST-elevation myocardial infarction (STEMI) patients using the VerifyNow P2Y12 function assay and reported individual patient data (from references^18–20^). Among the available P2Y12 inhibitors, only cangrelor ensures adequate periprocedural platelet inhibition in P2Y12 inhibitor-naïve patients.

Cangrelor provides rapid and profound platelet inhibition within a few minutes, outperforming crushed oral P2Y_12_ inhibitors in acute settings.^20,34^ The platelet Inhibition with Cangrelor and Crushed Ticagrelor in STEMI Patients Undergoing Primary Percutaneous Coronary Intervention (CANTIC) study was a prospective, randomized, double-blind, placebo-controlled study conducted in STEMI patients who received an initial loading dose of crushed ticagrelor and were randomized to treatment with either cangrelor or crushed ticagrelor.^20^ Patients receiving crushed ticagrelor showed a significantly higher platelet reactivity in the first 2 h compared with the cangrelor arm. Notably, ∼60% of patients in the ticagrelor arm (vs. 0% in the cangrelor arm) showed high platelet reactivity at the end of PCI.^20^ Similarly, cangrelor showed higher platelet inhibition than chewed and integral prasugrel 30 and 60 min after administration to P2Y_12_-naïve STEMI patients.^34^  *Figure 1* combines the pharmacological data obtained at different time points for prasugrel, ticagrelor (integral and crushed), clopidogrel, and cangrelor extracted from three independent studies (including CANTIC) that measured platelet reactivity in STEMI patients using the VerifyNow P2Y_12_ function assay.^18–20^

The relevance of achieving an adequate platelet inhibition at the time of PCI has been recently underlined by a sub-analysis of the Assessment of Loading with the P2Y_12_ Inhibitor Ticagrelor or Clopidogrel to Halt Ischaemic Events in Patients Undergoing Elective Coronary Stenting (ALPHEUS) trial. This sub-analysis investigated the impact of the time to PCI from loading with oral P2Y_12_ inhibitors (clopidogrel or ticagrelor) on periprocedural myocardial necrosis.^35^ Patients undergoing elective PCI (*N* = 1809) were divided into quartiles (*Q*) as a function of the P2Y_12_ inhibitor loading time before PCI. Patients grouped in the *Q*1 were loaded 0–0.2 h before the PCI, patients in the Q2 0.2–1.7 h before the PCI, patients in Q3 1.7–4.1 h before PCI, and those in the Q4 4.1–24 h before PCI. Patients in Q1, where the short time was not enough for the systemic absorption of the oral P2Y_12_ inhibitors, presented significantly higher rates of MI and major myocardial injury, which decreased in patients with earlier loading times. This study shows that loading P2Y_12_ inhibitor-naïve patients ‘on the table’ with oral P2Y_12_ inhibitors is insufficient to achieve adequate antithrombotic protection. The authors recommended a minimal waiting time between oral P2Y_12_ inhibitor loading and the PCI (e.g. 1 h) in P2Y_12_ inhibitor-naïve patients undergoing elective PCIs.

### Cangrelor compared with GPIIb/IIIa inhibitors

GPIIb/IIIa inhibitors are potent intravenous antiplatelets developed in the last decades of the last century. However, their use in ACS patients declined significantly with the introduction of more potent oral P2Y_12_ inhibitors, primarily due to the increased bleeding risk associated with these agents.

Cangrelor is often grouped with GPIIb/IIIa inhibitors under the category of ‘intravenous antiplatelets’ despite belonging to a different drug class and having a different mechanism of action.^36^ Cangrelor inhibits ADP-mediated platelet activation.^20^ In contrast, GPIIb/IIIa inhibitors act by blocking the GPIIb/IIIa receptor, preventing fibrinogen binding and, thereby, the final step of the platelet aggregation pathway. Without fibrinogen bridging, platelets cannot aggregate properly, which reduces thrombus formation very effectively.^37^ GPIIb/IIIa inhibitors induce a more potent antiplatelet effect than P2Y_12_ inhibitors.^34^ However, the high potency comes with increased risks of bleeding and thrombocytopenia.^38–42^ Of note, abciximab and eptifibatide are no longer available on the market, leaving tirofiban as the remaining GPIIb/IIIa.

Both cangrelor and GPIIb/IIIa inhibitors induce immediate platelet inhibition. However, cangrelor offers a faster offset, allowing for greater precision in managing antiplatelet therapy. For instance, in the event of bleeding, the effects of cangrelor begin to reverse immediately after discontinuation, with normal platelet function restored within 30–60 min. In contrast, GPIIb/IIIa inhibitors have a longer half-life, leading to a prolonged antiplatelet effect that persists for several hours (*Table 1*), which may pose challenges in cases requiring rapid reversal of platelet inhibition.

Cangrelor and GPIIb/IIIa inhibitors have not been compared in large randomized trials. A retrospective, single-centre study by Yerasi *et al.* compared the outcomes of ACS patients who received cangrelor (*N* = 478) or GPIIb/IIIa inhibitors (*N* = 1594) as adjunctive antiplatelet therapy during PCI.^40^ Patients in the cangrelor cohort had a lower rate of in-hospital major bleedings (1.7% vs. 5.1%, *P* = 0.001) and lower rates of vascular complication than those in the GPIIb/IIIa cohort (1.5% vs. 3.2%, *P* = 0.04). At 30 days, all-cause mortality, cardiac death, and ST rates did not differ between groups. However, significantly lower rates of MI (0% vs. 1.8%, *P* = 0.03) and ischaemia-driven revascularization (IDR, 0% vs. 1.9%, *P* = 0.03) were noted in the cangrelor group.^40^ An exploratory analysis of the pooled data from the registrational Cangrelor Versus Standard Therapy to Achieve Optimal Management of Platelet Inhibition (CHAMPION) trials compared patients treated with clopidogrel (or placebo) and routine GPIIb/IIIa inhibitors (*N* = 1211) vs. patients treated with cangrelor (*N* = 10 929, no GPIIb/IIIa inhibitors). Although there were no differences in ischaemic outcomes, major and minor bleedings [according to the Thrombolysis in Myocardial Infarction (TIMI) criteria] were increased in patients receiving GPIIb/IIIa inhibitors.^43^

Finally, a post-hoc analysis of the CHAMPION programme did not find an increased risk of acquired thrombocytopenia associated with cangrelor. However, GPIIb/IIIa inhibitor use was the strongest independent predictor of acquired thrombocytopenia (OR, 2.93; 95% CI, 2.15–3.97; *P* < 0.0001). Of note, the adjusted rate of major adverse cardiovascular events (MACE) and major bleedings were significantly higher in patients who developed thrombocytopenia.^44^

## Practical aspects of cangrelor

### Approval, dosage, and guidelines recommendations

Cangrelor was approved by the European Medicines Agency (EMA) and the Food and Drug Administration (FDA) in 2015.^45,46^ Cangrelor is presented as a powder for reconstitution to be administered as an intravenous bolus (30 µg/kg in one minute) prior to PCI followed by a 4 µg/kg/min intravenous infusion for at least two hours or the duration of the procedure, whichever is longer. In selected cases (i.e. patients treated with opiates), cangrelor infusion can be continued for up to 4 h at the discretion of the physician.^45^ Prolonged cangrelor infusions (>6 h and up to 7 days) at a delivery rate of 0.75 µg/kg/min have been used to bridge patients from PCI to CABG surgery or to cover the gap until the onset of action of oral P2Y_12_ inhibitors in comatose patients managed with therapeutic hypothermia.^27,47^

Cangrelor holds a Class IIb recommendation in both the 2023 ESC Guidelines and the 2025 ACC/AHA/ACEP/NAEMSP/SCAI Guideline.^1,2^ It was developed when clopidogrel was the standard of care, but its safety and efficacy compared to ticagrelor or prasugrel—both of which carry Class Ia recommendations—have not been directly established in randomized trials for ischaemic event reduction. However, according to the guidelines, cangrelor may be considered in clinical scenarios where oral P2Y_12_ inhibitor absorption is impaired or not feasible, as well as in patients requiring early CABG surgery after PCI.^1^

### Transitioning from cangrelor to oral P2Y_12_ inhibitors

The primary goal of transitioning from cangrelor to oral P2Y_12_ inhibitors is to ensure effective and continuous platelet inhibition from the initiation of cangrelor administration until the antiplatelet effect of the oral P2Y_12_ inhibitors is achieved. This strategy is critical for preventing gaps in platelet inhibition, which could increase the risk of acute stent thrombosis. However, the transition process differs between ticagrelor, clopidogrel, and prasugrel due to differences in the P2Y_12_ receptor binding site, onset and offset of action, and half-life.

Ticagrelor is the most versatile option for transitioning from cangrelor to oral P2Y_12_ inhibition, and it can be administered before, at the start, or during cangrelor infusion.^2,27^ Ticagrelor binds to the P2Y_12_ receptor through an allosteric, non-competitive mechanism and, therefore, does not inhibit cangrelor binding [i.e. no drug–drug interaction (DDI)].^28^ In addition, ticagrelor's half-life ranges between 6 and 10 h.^27^ Therefore, even in pre-treated patients, by the end of the cangrelor infusion (i.e. 120 min), ticagrelor's systemic bioavailability remains sufficiently high to ensure adequate platelet inhibition as cangrelor's effect gradually offsets over 30–60 min. Giving ticagrelor at the start of the cangrelor infusion is the most effective strategy, considering that ticagrelor achieves the median maximum concentration in blood 1.5–3 h after loading.^48^ The prospective, randomized, double-blind Switching Antiplatelet (SWAP)-5 study demonstrated that the use of cangrelor in patients pre-treated with ticagrelor achieves enhanced platelet inhibition in coronary artery disease patients without DDIs.^49^ Non-surprisingly, real-world studies indicate that ticagrelor is the preferred oral P2Y_12_ inhibitor after cangrelor (73%–89%).^22,50–52^

On the contrary, the transition from cangrelor to thienopyridines (clopidogrel and prasugrel) requires a careful approach because there is a competitive interaction between the active metabolites of clopidogrel and prasugrel and cangrelor to bind the P2Y_12_ receptor. Clopidogrel's active metabolite has a short half-life (∼30 min). If it enters the systemic circulation while P2Y_12_ receptors are still occupied by cangrelor, it will be rapidly degraded, resulting in inadequate platelet inhibition and an increased risk of thrombotic complications after cangrelor discontinuation.^53^ Therefore, clopidogrel must be administered at the end of the cangrelor infusion.^27,36^ Notably, however, this approach may create a gap in platelet inhibition due to cangrelor's rapid offset (30–60 min) and clopidogrel's delayed systemic absorption (>2 h), potentially increasing thrombotic risk.

Prasugrel's active metabolite also shows DDI with cangrelor.^28^ However, it has a slightly longer half-life than clopidogrel, which prolongs its systemic circulation. A randomized trial showed adequate platelet inhibition with a loading dose of prasugrel given concomitantly with the cangrelor bolus at the start of PCI.^54^ Nevertheless, the recent SWAP-6 study has reported a DDI between cangrelor and prasugrel, demonstrating that their concomitant administration significantly increased platelet reactivity 1 h after stopping the cangrelor infusion.^55^ The PK analysis of the SWAP-6 study revealed low levels of prasugrel's active metabolite at the end of cangrelor infusion, providing compelling evidence of this interaction. Therefore, prasugrel should be administered at the end of the cangrelor infusion to avoid DDIs.

**Figure 2:**
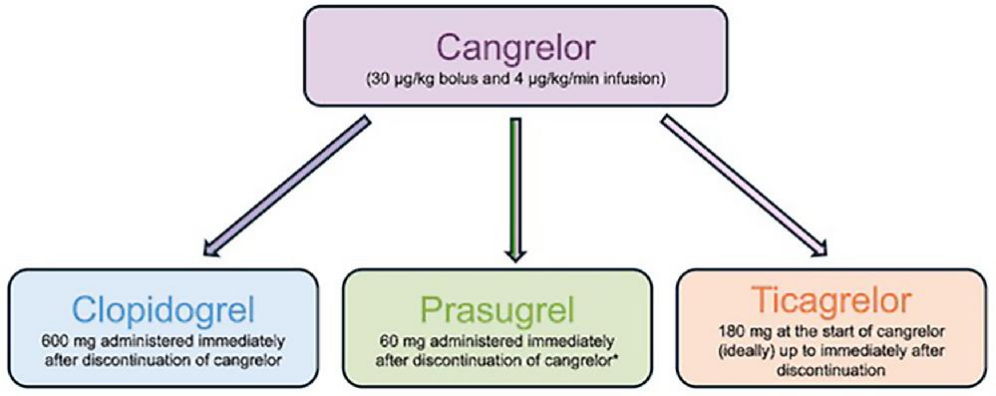
Transition from intravenous to oral P2Y12 inhibitors according to the international expert consensus on switching platelet P2Y12 receptor–inhibiting therapies. The asterisk symbol denotes that prasugrel can also be administered 30 min before the end of cangrelor discontinuation. Adapted from Angiolillo *et al.* (reference^27^).

The 2023 ESC Guidelines and the International Expert Consensus on Switching Platelet P2Y_12_ Receptor–Inhibiting Therapies recommend administering a loading dose of thienopyridines (clopidogrel or prasugrel) immediately after discontinuation of cangrelor infusion (*Figure 2*).^2,27^ Prasugrel may also be administered 30 min before the cangrelor infusion stops.^2^ On the other hand, a loading dose of ticagrelor can be administered before, during, or immediately after cangrelor infusion. However, earlier administration (e.g. at the time of PCI) should be considered.

### Cangrelor in patients pre-treated with oral P2Y_12_ inhibitors

Although cangrelor is indicated for P2Y_12_ inhibitor-naïve patients, it is frequently used in those already pre-treated with oral P2Y_12_ inhibitors.^56,57^ In the Cath Lab, operators may be unaware of whether a patient has received upstream oral P2Y_12_ inhibitors. Additionally, they may opt for cangrelor in cases where systemic absorption is compromised or in acute settings where there is not enough time for the onset of oral antiplatelets.

Cangrelor has an additive antiplatelet effect in patients receiving upstream oral P2Y_12_ inhibitors.^58–60^ However, randomized clinical trials have not established the safety of this approach. The most recent analysis of the Cangrelor in Acute Myocardial Infarction: Effectiveness and Outcomes (CAMEO) registry did not find significant differences in the bleeding rates between patients treated with cangrelor with and without upstream P2Y_12_ inhibitor exposure.^57^ Similarly, the Swedish Coronary Angiography and Angioplasty (SCAAR) registry did not report excess bleeding in the cangrelor cohort, even though one-third of the patients in the cangrelor cohort had been pre-treated with ticagrelor.^56^ It is worth noting that in the SCAAR and CAMEO registries, cangrelor was mostly used in patients who had been pre-treated with ticagrelor (>95%). This suggests that Cath Lab operators are aware of the lack of DDIs between ticagrelor and cangrelor and are therefore confident combining cangrelor with ticagrelor to ensure adequate antithrombotic.

## Cangrelor use in contemporary PCI: insights from real-world studies

Cangrelor's approval was based on comprehensive data from the CHAMPION programme involving approximately 25 000 patients across three randomized controlled trials: CHAMPION PCI, CHAMPION PLATFORM, and CHAMPION PHOENIX.^61–63^ A pooled analysis with patient-level data from the 3 CHAMPION trials showed a significant reduction of 19% in the primary composite outcome (death, MI, IDR, and ST at 48 h) with cangrelor use compared with clopidogrel (OR, 0.81; 95% CI, 0.71–0.91; *P* = 0.0007), without severe or life-threatening bleeding (0.2% in both groups, according to Global Use of Strategies to Open Occluded Coronary Arteries GUSTO) across the whole ACS spectrum.^64^

CHAMPION PCI and CHAMPION PLATFORM were planned and performed before 2008, at this time neither prasugrel nor ticagrelor have been commercially available. The CHAMPION PHOENIX trial was performed between September 2010 and October 2012. The first guideline that recommended prasugrel and ticagrelor over clopidogrel was the 2011 ESC NSTE-ACS guidelines, published after the protocol of the CHAMPION PHOENIX had been written and the trial had already started. Still, it could be argued that prasugrel and ticagrelor would have been more appropriate comparators in patients with acute coronary syndromes, unfortunately, such large-scale randomized comparisons are not available.

Real-world data provide an important complementary source of information to fill this evidence gap, offering insights into the contemporary adoption and clinical utility of cangrelor. Real-world studies on cangrelor have involved over 6000 patients across various geographies and have primarily focused on its safety, particularly concerning bleeding (*Table 2*).^22,50–52,56,57,65,66^ These studies highlight that cangrelor is mainly used in high-risk ACS patients, with STEMI cases comprising 48%–98% of treated individuals. Notably, approximately 10% of cangrelor-treated patients in real-world studies present with CS and CA, a population excluded from the CHAMPION trials.

**Table 2:** Overview of selected real-world studies reporting cangrelor use after its approval in 2015

	Year/Country	Cangrelor-treated patients	STEMI Population	CS or CA patients?	Any Bleeding	MACE^a^	GPIIb/IIIa inhibitor use	Observations
CHAMPION POOLED^64^	2013/International	*N* = 12 475	11.3%	No	17.5%	5.3%	12.4%	Cangrelor reduced the odds of the primary outcome by 19% (*P* = 0.0007) and ST by 41% (*P* = 0.0008) without increased severe or life-threatening bleeding by GUSTO at 48 h.
Vaduganathan *et al.*^52^	2017/USA	*N* = 100	49%	Yes (16% CS and 9% CA)	18%	∼5%	0%	Bleeding was defined by GUSTO at 48 h. Cangrelor was used in a higher-risk cohort than represented in the CHAMPION programme.
SCAAR Registry^56^	2019/Sweden	*N* = 915	98.2%	Yes (17.8% CA)	2.3%	15.8%	4.2%	Bleeding was assessed by ARC at 30 days. Cangrelor was used nearly exclusively in STEMI patients (>98%). 33% of patients were pre-treated with oral P2Y_12_ inhibitors (mostly ticagrelor) before cangrelor.
DUTCH Registry^66^	2021/The Netherlands	*N* = 250	–	Yes (8% CA)	7.6%	0.8%	–	Bleeding was assessed by BARC at 48 h. Most bleedings (7.2%) were minor (type 2). Low incidence of ischaemic events in the first 48h: Death 0.4% and ST and MI 0%.
CAMEO Registry I^50^	2022/USA	*N* = 819	57.3%	Yes (9% CS and 3% CA)	6%	9.1%	2%	Most patients (85.3%) transitioned from cangrelor to ticagrelor. Just 27% of patients received cangrelor as per the FDA label. Patients receiving cangrelor as per label had lower (non-significant) bleeding and MACE (death, MI, IDR, and ST) rates.
ARCANGELO Registry^22,67^	2023/Italy	*N* = 995	60%	Yes (2% CS)	5.23%	1.4%	2.6%	Bleeding was assessed by BARC at 30 days post-PCI. MACE (death, MI, IDR, and ST) rate within 30 days post-PCI was 1.4%. Most patients (73.4%) transitioned to ticagrelor.
M.O.Ca. Registry^65^	2023/Italy	*N* = 198	58.1%	YES (12.6% CA)	2.5%	–	1.5%	Cangrelor was mainly used in patients with high-risk clinical features and a tendency to high bleeding risk. In a matched analysis, cangrelor significantly reduced ST compared to the oral P2Y_12_ cohort (*P* = 0.03)
Thim *et al*.^51^	2023/Denmark	*N* = 991	73%	Yes (12% CA)	2.9%	∼2.8%	2.2%	Cangrelor was mostly used in acute PCI settings (88%). Bleeding assessment included BARC types 3 and 5. Four of the six fatal bleeding events occurred in patients resuscitated after out-of-hospital cardiac arrest.
CAN-SHOCK study^68^	2023 Germany and Austria	*N* = 303	–	Yes (49% CA, 14% CS, and 34 CA and CS)	11.2%	∼23%	–	N = 118 patients of the CAN-SHOCK population were matched with the N = 118 from the CULPRIT-SHOCK population (i.e. managed with oral P2Y12 inhibitors). The comparison showed a numerically lower rate of ischaemic and bleeding events in the CAN-SHOCK cohort, with a trend towards lower cardiovascular death.
CAMEO Registry II^57^	2024/USA	*N* = 1802	57.5%	Yes (8% CS and 3% CA)	8.1%	–	3%	Bleeding events were defined as any event associated with a haemoglobin drop ≥3 gm/dL, requiring blood transfusion, or any bleeding event requiring an intervention or surgery. There was no statistically significant difference in bleeding among cangrelor-treated patients with and without upstream oral P2Y12 inhibitor exposure.

The pooled analysis of the three CHAMPION trials is included for comparison.

ARC, academic research consortium; BARC, bleeding academic research consortium; CA, cardiac arrest; CS, cardiogenic shock; FDA, Food and Drug Administration; GPIIb/IIIa, glycoprotein IIb/IIIa; GUSTO, Global Use of Strategies to Open Occluded Coronary Arteries; IDR, ischaemia-driven revascularization; MACE, major adverse cardiovascular events; MI, myocardial infarction; PCI, percutaneous coronary; ST, stent thrombosis; STEMI, ST-segment elevation myocardial infarction.

^a^MACE include death, myocardial infarction, coronary revascularization, and stroke. In some studies, the MACE rate was not directly reported but was instead estimated based on the incidence of its individual components. It is important to note that the assessment timeframe for MACE varies across studies, which may impact comparability.

The SCAAR registry reported the first 2 years of routine use of cangrelor in Sweden (2016–2018).^56^ SCAAR registered *N* = 899 patients treated with cangrelor and *N* = 4614 treated with oral P2Y_12_ inhibitors. Remarkably, cangrelor was used almost exclusively in STEMI patients (N = 899, 98%), including 17.8% of STEMI patients presenting with CA. Notably, two-thirds (160/235) of all CA patients in the SCAAR Registry were treated with cangrelor, indicating a preference for cangrelor over oral P2Y_12_ inhibitors in this high-risk setting. Despite cangrelor being used more frequently in high-risk patients (i.e. left main, thrombus aspiration, and CS), all-cause mortality at 30 days and ST did not differ from the cohort treated with oral P2Y_12_ inhibitors (*N* = 4215). No major bleedings were recorded in the cangrelor cohort, and bleedings within hospital stay did not differ between cangrelor and non-cangrelor cohorts (2.3% and 2.8%, respectively). GPIIb/IIIa inhibitor use during PCI was lower in the cangrelor cohort (4.2% vs. 10.4%). Notably, one-third of patients in the cangrelor cohort had been pre-treated with ticagrelor despite cangrelor being indicated in oral P2Y_12_-naïve patients.

The CAMEO registry is an ongoing registry of 12 US centres enrolling patients since 2019. Two reports of the CAMEO registry have been published to date.^50,57^ The first investigated the transition from cangrelor to oral P2Y_12_ inhibitors after PCI, revealing that most patients switched to ticagrelor (85.3%), followed by clopidogrel (9.5%), and prasugrel (5.2%).^50^ Of note, just 27.3% of patients in CAMEO received cangrelor as per the FDA label: 38.3% of cangrelor-treated patients received an infusion of <2 h duration, and about 17% of patients had a gap >1 h between cangrelor cessation and oral P2Y_12_ inhibitor loading. Although ischaemic and bleeding events did not significantly differ between patients who received cangrelor as per label and those who did not, bleeding (4.5% vs. 6.6%) and MACE (6.3% and 10%) were numerically higher in patients who received cangrelor out of the label-established indications.

The second CAMEO report investigated the bleeding risk of ACS patients who were given upstream oral P2Y_12_ inhibitors.^57^ In total, *N* = 298 patients (16%) were pre-treated in-hospital with oral P2Y_12_ inhibitors (mostly with ticagrelor >90%). Among these, 33.8% started cangrelor within 1 h, 34.4% between 1 and 3 h, and 31.8% after more than 3 h. Bleeding rates did not differ between patients receiving upstream oral P2Y_12_ inhibitors and oral P2Y_12_-naïve patients (6.5% vs. 8.8%, adjusted OR 0.62; 95% CI, 0.38–1.01). Similarly, there were no significant differences in bleeding rates according to the timing of pre-treatment. However, the bleeding rate was slightly higher in patients who received cangrelor 1–3 h after pre-treatment (10.7%) than in those who received cangrelor within one hour (5%) or after more than 3 h (3.2%).

The Italian Prospective Study on Cangrelor (ARCANGELO) was a multicentre, post-approval study requested by the EMA to evaluate the safety of cangrelor when used according to label-directed transition to oral P2Y_12_ inhibitors (*N* = 995).^22,67^ The bleeding rate with cangrelor at 30 days post-PCI was 5.2% (primary outcome), with most bleedings (94.2%) classified as mild, according to the Bleeding Academic Research Consortium (BARC) criteria (type I and II). The incidence of MACE was 1.4%, with 70% of the events occurring within the first 48 h post-PCI. GPIIb/IIIa inhibitors were administered in 2.6% of the patients, almost exclusively in STEMI patients with thrombotic complications (96%). Contrary to CAMEO, cangrelor was administered according to the label indications. Notably, 98.3% of patients received a cangrelor bolus for 2–4 h. The reasons for choosing cangrelor were urgency of PCI (89.2%), difficulty in swallowing and nausea/vomiting (6.7%), CS or intubation (3.6%), and possible cardiac surgery (1.4%). After PCI, most patients were transitioned to ticagrelor (73.4%), followed by clopidogrel (13.9%), and prasugrel (12.8%).

Two recent studies have also reported the use of cangrelor in challenging PCI settings.^51,65^ Thim *et al.* reported the use of cangrelor in *N* = 991 patients at Aarhus Hospital (Denmark) between 2016 and 2018.^51^ Cangrelor was primarily used in acute PCIs (87%), predominantly in STEMI patients (73%), but also in patients presenting with CA (12%). Rates of acute MI, ST, and ischaemic stroke were low (all 0.2%), and BARC type 3 and 5 bleedings (primary safety outcome) occurred in 2.4% of patients. Notably, all six patients with fatal bleeding underwent acute procedures, and four out of six occurred in patients resuscitated after out-of-hospital CA (OHCA).

The M.O.Ca. Registry collected data on *N* = 696 consecutive ACS patients between 2019 and 2021 in two Italian centres.^65^  *N* = 198 patients were included in the cangrelor cohort, which was notably unbalanced compared to the oral P2Y_12_ inhibitor cohort. Cangrelor was predominantly used in patients with high-risk clinical features and an increased bleeding tendency. In addition, patients in the cangrelor cohort underwent significantly more extensive and complex revascularization procedures. After propensity score matching to adjust for differences in baseline and procedural characteristics, *N* = 198 patients from each cohort were compared. The matched cohorts showed similar bleeding rates (cangrelor 2.8% vs. oral P2Y_12_ inhibitors 3.4%), but the incidence of definite ST was significantly lower in the cangrelor cohort (0% vs. 2.8%).

In summary, real-world studies confirm the safety of cangrelor in terms of bleeding but highlight key differences between its contemporary use and the CHAMPION trials: (i) cangrelor is primarily used in high-risk patients in the real world, predominantly in STEMI patients, including those presenting with CS and CA; (ii) most patients transition to ticagrelor after PCI, rather than other P2Y_12_ inhibitors; (iii) although cangrelor is indicated for P2Y_12_ inhibitor-naïve patients, its use in patients pre-treated patients is not uncommon; (iv) bleeding rates and GPIIb/IIIa inhibitor use are generally lower in real-world studies than in the CHAMPION trials. Nevertheless, these findings must be interpreted with great caution, considering evolving guidelines and scientific advancements in PCI since CHAMPION trials began nearly two decades ago.

## Cangrelor in patients presenting with cardiogenic shock and cardiac arrest

The incidence of CS in patients with acute MI ranges from 3% to 13% and is associated with high in-hospital mortality (>40%).^69^ Approximately 50% of MI-related CS patients experience a CA.^70^ Early revascularization of the culprit lesion has been shown to reduce mortality and is a beneficial approach in the management of CS settings of MI.^71^

However, achieving adequate platelet inhibition with oral DAPT is very challenging in CS and CA settings because several factors that compromise the systemic absorption of oral antiplatelets converge in these patients.^19,31,32,72,73^ The risk of bleeding is also accentuated in CS and CA patients, which requires careful consideration of the antiplatelet strategy. In such acute settings, a parenteral antiplatelet strategy with intravenous ASA and cangrelor should be considered as the only possibility of providing a timely DAPT.

**Figure 3:**
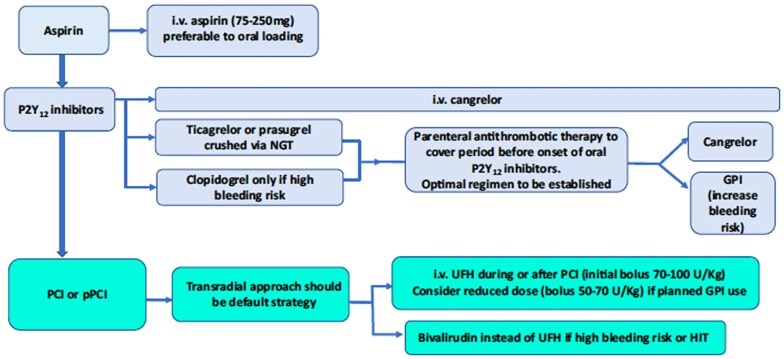
Suggested antithrombotic therapy pathway in patients with cardiogenic shock or out-of-hospital cardiac arrest according to the joint position paper from the European Society of Cardiology (ESC) working group on thrombosis, in association with the acute cardiovascular care association (ACCA) and European association of percutaneous cardiovascular interventions (EAPCI). GPI, Glycoprotein IIb/IIIa inhibitor; HIT, heparin-induced thrombocytopenia; NGT, nasogastric tube; PCI, percutaneous coronary intervention; pPCI, primary percutaneous coronary intervention; UFH, unfractionated heparin. From Gorog *et al.* (reference^72^).

A joint position paper on antithrombotic therapy in patients with ACS complicated by CS or OHCA from the ESC Working Group on Thrombosis, in association with the Acute Cardiovascular Care Association (ACCA) and the European Association of Percutaneous Cardiovascular Interventions (EAPCI) acknowledges the benefits of cangrelor in critically ill patients, proposing its use for the initial antithrombotic therapy in patients with CS or OHCA.^72^ The position paper suggests that cangrelor can be used as the default periprocedural P2Y_12_ inhibitor or administered concomitantly with oral P2Y_12_ inhibitors to cover the period until their onset of action (*Figure 3*).^72^ The upstream use of GPIIb/IIIa has also been proposed in CS and CA settings as they may improve ischaemic outcomes and can also be used to bridge the gap until the onset of oral P2Y_12_ inhibitors.^74–76^ Nevertheless, cangrelor is the preferred antiplatelet agent in CS and CA patients due to its lower bleeding risk, while GPIIb/IIIa inhibitors should be reserved for bailout situations.^72^

Several clinical studies have reported the use of cangrelor in CS and CA patients.^17,22,47,50–52,56,57,65,66,68,77,78^ In a recent study, cangrelor safely induced immediate and profound platelet inhibition in comatose survivors of OHCA undergoing PCI and transient temperature management.^17^ After intensive care admission, all patients received intravenous ASA and a loading dose of ticagrelor (180 mg) and were randomized to cangrelor or control groups. Cangrelor provided superior platelet inhibition at 1 h (30 ± 45 vs. 221 ± 45 PRU; *P* < 0.001) and 3 h (24 ± 36 vs. 180 ± 67 PRU; *P* < 0.001), without significant differences at 5 and 8 h between groups, thus bridging the gap until ticagrelor's onset of action. In another study, Prüller *et al.* demonstrated a more potent platelet inhibition with cangrelor than oral P2Y_12_ inhibitors in resuscitated patients without increasing major or minor bleedings.^47^

A global, multicentre study by Droppa *et al.*^77^ compared 88 matched pairs of cangrelor-treated CS patients with patients managed with oral P2Y_12_ inhibition from the Intra-aortic Balloon Pump in Cardiogenic Shock II (IABP-SHOCK II) trial;^79^ 30-day and 12-month mortality rates were 29.5% and 34.1% in cangrelor-treated patients and 36.4% and 47.1% in the control group (*P* = 0.34 and *P* = 0.08, respectively), and moderate and severe bleeding rates were 26% with cangrelor and 19.3% with oral P2Y_12_ inhibitors. Notably, TIMI flow ≥1 grade during PCI was achieved more frequently with cangrelor (92.9% vs. 81.2%, *P* = 0.02).

**Figure 4:**
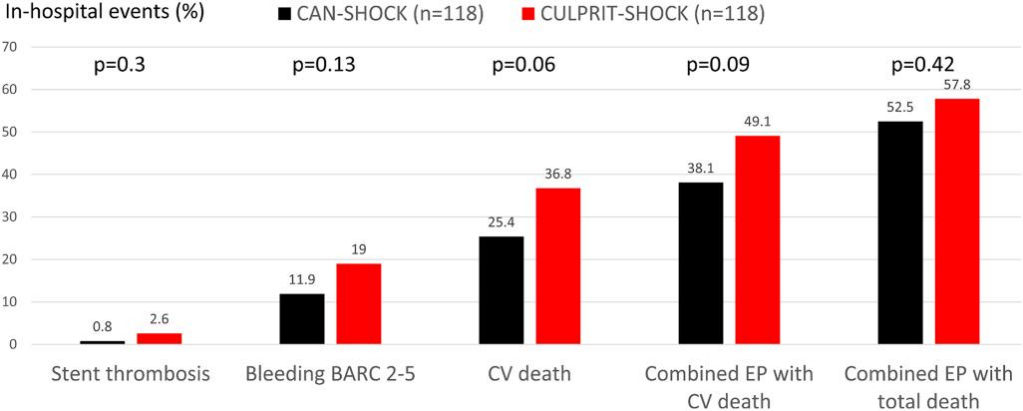
Comparison of in-hospital events in a matched comparison between 118 patients included in the CAN-SHOCK trial treated with cangrelor and 118 patients included in the CULPRIT-SHOCK trial treated with oral P2Y12 inhibitors. Combined Endpoint (EP): death, stent thrombosis, re-myocardial infarction, stroke, and Bleeding Academic Research Consortium (BARC) 2–5 bleeding. CV, cardiovascular. From Zeymer *et al.* (reference ^68^).

We have recently reported a series of *N* = 303 cangrelor-treated CS and CA patients undergoing PCI from the CAN-SHOCK trial.^68^ ST and myocardial re-infarction within the first 48 h (primary outcome) were reported in two (0.7%) patients. BARC type 1–3 bleedings occurred in 11.2% of patients and fatal bleedings (BARC 5) in 3.3%. The total in-hospital mortality rate was 41.6%, with cardiac mortality accounting for 21.8%. The patients of the CAN-SHOCK trial were matched with those from the Culprit Lesion Only PCI vs. Multi-vessel PCI in Cardiogenic Shock (CULPRIT-SHOCK) trial (i.e. managed with oral P2Y_12_ inhibitors),^71^ generating two matched cohorts with *N* = 118 patients each. The comparison showed a numerically lower rate of ischaemic and bleeding events in the CAN-SHOCK cohort, with a trend towards lower cardiovascular death (*Figure 4*).

Although non-significant, the trends towards lower mortality reported by Droppa *et al.* and the CAN-SHOCK trial are encouraging and set the rationale for investigating the safety and efficacy of cangrelor in CS and CA patients in randomized clinical trials. The ongoing multicentre, randomized, double-blind Dual Antiplatelet Therapy for Shock Patients with Acute Myocardial Infarction (DAPT-SHOCK) trial (NCT03551964) is evaluating intravenous cangrelor vs. oral ticagrelor in patients with acute MI complicated by CS.^80^ In this placebo-controlled trial about 550 patients with infarct-related CS undergoing PCI will be enrolled. The primary clinical outcome is a composite of death, myocardial infarction, and stroke until day 30, the primary safety endpoint BARC bleeding complications. The results of this trial are expected to clarify cangrelor's role in the management of CS patients and potentially shape future treatment guidelines.

## Conclusions

Cangrelor offers advantages in acute, high-risk PCI settings. Its rapid onset of action provides profound platelet inhibition within 5 min, a level of immediacy that oral P2Y_12_ cannot achieve in patients undergoing urgent PCIs. Compared to GPIIb/IIIa inhibitors, cangrelor is associated with a lower risk of bleeding and thrombocytopenia, while its faster offset of action allows for greater precision in managing antiplatelet therapy. Unlike its use in registrational studies, where it was used in a limited number of STEMI patients, real-world studies indicate that cangrelor is predominantly used in STEMI cases. A large-scale trial in STEMI with contemporary PCI techniques comparing Cangrelor with the newer more potent oral agents prasugrel and ticagrelor would be helpful to define its role in this setting. Cangrelor may play an important role in STEMI cases presenting with CS and CA, where multiple factors that hinder the systemic absorption of oral P2Y_12_ inhibitors converge. The outcomes of preliminary studies using cangrelor in CS patients have been encouraging, providing the rationale for the ongoing DAPT-SHOCK randomized controlled trial, which is comparing intravenous cangrelor with oral ticagrelor in patients with acute MI complicated by CS.

Following PCI, the transition from cangrelor to oral P2Y_12_ inhibitors requires a strategy tailored to the specific agent. Thienopyridines (clopidogrel and prasugrel) exhibit a DDI with cangrelor and must be administered towards the end of cangrelor infusion to ensure effective platelet inhibition. Ticagrelor, on the other hand, is the most versatile option for transitioning from cangrelor to oral P2Y_12_ inhibition, as there is no DDI between the two agents. Therefore, ticagrelor can be administered before, at the start, or during cangrelor infusion, offering greater flexibility in antiplatelet management.

## Data Availability

No new data were generated or analysed in support of this research.
